# The role of areas MT+/V5 and SPOC in spatial and temporal control of manual interception: an rTMS study

**DOI:** 10.3389/fnbeh.2013.00015

**Published:** 2013-03-05

**Authors:** Joost C. Dessing, Michael Vesia, J. Douglas Crawford

**Affiliations:** ^1^Centre for Vision Research, York UniversityToronto, ON, Canada; ^2^Canadian Action and Perception NetworkToronto, ON, Canada; ^3^School of Psychology, Queen's University BelfastBelfast, Northern Ireland, UK; ^4^Brain Sciences Research Program, Sunnybrook Research InstituteToronto, ON, Canada; ^5^Department of Kinesiology, University of WaterlooWaterloo, ON, Canada; ^6^Department of Medicine, Division of Neurology and Krembil Neuroscience Centre, Toronto Western Research Institute, University of TorontoToronto, ON, Canada; ^7^Neuroscience Graduate Diploma Program and Departments of Psychology, Biology, Kinesiology and Health Sciences, York UniversityToronto, ON, Canada

**Keywords:** interception, rTMS, SPOC, MT+, spatial behavior, timing of action

## Abstract

Manual interception, such as catching or hitting an approaching ball, requires the hand to contact a moving object at the right location and at the right time. Many studies have examined the neural mechanisms underlying the spatial aspects of goal-directed reaching, but the neural basis of the spatial and temporal aspects of manual interception are largely unknown. Here, we used repetitive transcranial magnetic stimulation (rTMS) to investigate the role of the human middle temporal visual motion area (MT+/V5) and superior parieto-occipital cortex (SPOC) in the spatial and temporal control of manual interception. Participants were required to reach-to-intercept a downward moving visual target that followed an unpredictably curved trajectory, presented on a screen in the vertical plane. We found that rTMS to MT+/V5 influenced interceptive timing and positioning, whereas rTMS to SPOC only tended to increase the spatial variance in reach end points for selected target trajectories. These findings are consistent with theories arguing that distinct neural mechanisms contribute to spatial, temporal, and spatiotemporal control of manual interception.

## Introduction

In most everyday situations, human interactions with objects in the surrounding environment are inherently dynamic. For example, during manual interception tasks such as catching or hitting an approaching ball, the object of interest or the human is usually in motion. Although interception has been in the forefront of ecological psychology (Michaels et al., 2006), it has only recently garnered interest within the neuroscience community (Merchant et al., 2009; Zago et al., 2009). Neurophysiological studies on manual interception have focused predominantly on temporal performance, namely how the brain controls interceptive timing. Interception, however, also requires accurate spatiotemporal mechanisms (Peper et al., 1994; Dessing et al., 2002, 2005, 2009). Yet, the neural bases of both mechanisms remain poorly understood in the human. Here, our aim was to determine specific neuroanatomical regions involved in spatial and temporal control of interceptive reaching.

Neuroimaging studies in humans have shown that a distributed parietofrontal cortical network is involved in interception (Senot et al., 2008; Tan et al., 2009; Tombini et al., 2009). Single-unit recordings in monkeys have implicated parietal area 7a (Merchant et al., 2004a) and the primary motor cortex, with the latter not only showing movement execution-related activity, but also target motion-dependent activity (Port et al., 2001; Merchant et al., 2004a). Perhaps less surprisingly, because of its sensitivity to moving targets, the middle temporal area (MT+/V5) has been implicated in the temporal control of simple forms of interception (Schenk et al., 2005; Bosco et al., 2008; see also Field and Wann, 2005). While MT+/V5 also likely plays a role in motion extrapolation (Olson et al., 2004; Boulinguez et al., 2009; Kaas et al., 2010), to date this has never been explicitly investigated during interception in humans (but see Ilg and Schumann, 2007).

Numerous studies have examined the spatial control of reaching to stationary targets (Soechting and Flanders, 1992; Crawford et al., 2004, 2011). In monkeys, the caudal part of the superior parietal lobule (SPL) plays a critical role in the sensorimotor transformations required for planning and executing movements directed to external stimuli (Galletti et al., 1997; Buneo and Andersen, 2006). Interestingly, surrounding areas within the intraparietal and parieto-occipital sulci also have been implicated in the encoding of visual motion (Colby et al., 1993; Merchant et al., 2004b; Pitzalis et al., 2010; Fanini and Assad, 2009). In humans, a corresponding parietal region for reach that encodes the goal position in visual coordinates has been identified in the superior parieto-occipital cortex (SPOC) (Astafiev et al., 2003; Connolly et al., 2003; Prado et al., 2005; Fernandez-Ruiz et al., 2007; Filimon et al., 2009; Gallivan et al., 2009, 2011; Bernier and Grafton, 2010; Cavina-Pratesi et al., 2010; Vesia et al., 2010; *for a recent review see* Vesia and Crawford, 2012). Despite considerable evidence for a predominant role of caudal SPL for visual reaching to stationary targets in the posterior parietal cortex for both monkey and human, it remains to be shown whether similar neural mechanisms control interceptive reaching.

To probe the specific brain mechanisms in the spatial and temporal control of manual interception, we applied repetitive transcranial magnetic stimulation (rTMS) to MT+/V5 and SPOC, while participants performed a screen-based manual interception task that involved an hand movement to a target moving in a downward direction on a screen along an unpredictably curved trajectory. We hypothesized that disruption of neural activity in MT+/V5 would interfere not only with temporal control, but also with spatial control in manual interception. We also hypothesized that SPOC would be involved in the spatial control of manual interception. Although the observed rTMS effects proved to be subtle and occurred for selected target trajectories, they were generally consistent with these predictions.

## Materials and methods

### Participants

Seven right-handed (Oldfield, 1971) volunteers participated in all experiments (five males, two females, age range 21–34 years). All participants had normal or corrected-to-normal vision. Before participating, they provided written informed consent and completed a screening form for contra-indications to magnetic resonance imaging (MRI) and TMS (Keel et al., 2001; Rossi et al., 2009); all participants had no known risk factors for MRI or TMS. No side effects attributable to TMS were reported by any of the participants, other than discomfort associated with stimulation of MT+/V5 (see below). All procedures were approved by the York University Human Participant Review Subcommittee and adhered to the Declaration of Helsinki.

### Experimental set-up

During the experiment, participants were seated on a height-adjustable chair with their heads immobilized using a head-rest and a personalized bite-bar made out of dental compound. They were facing a 21 inch Dell Trinitron Cathode Ray Tube (CRT) monitor (85 Hz) positioned on a table in front of them. Their elbow was resting on a box and their right index finger pressing a button (30 cm in front of and 6 cm below the screen center). The bite-bar ensured that the cyclopean eye position on average was at the height of screen center, 50 cm from the screen surface. The experiment took place in complete darkness; stimuli on the CRT screen were the only light sources. To prevent the hand's silhouette from appearing when it was held in front of the screen and the screen edges from being discernable, two layers of 95% light blocking coating (Gila, St. Louis, MO) were applied on the CRT screen surface. Stimuli and events were controlled using a custom program generated using the Psychophysics Toolbox for Matlab (Version 3, Brainard, 1997).

During the main experiment, eye and finger movements were recorded. An Eyelink II camera (SR Research Ltd., Mississauga, ON, Canada) was mounted to the bite-bar, allowing registration of the right eye-in-head orientation (sample rate: 250 Hz). The camera was detached from its head-band to allow the positioning of the TMS coil anywhere on the skull without any physical restrictions. Movement of the right index finger was recorded using an Optotrak 3020 camera system (sample rate: 200 Hz; Northern Digital, Inc., Waterloo, ON, Canada; see “Behavioral Analyses”).

### Neuronavigation

To identify loci of interest and monitor the TMS coil position in real-time, we used frameless stereotaxic neuronavigation (Brain Voyager TMS Neuronavigator; Brain Innovation, Maastricht, The Netherlands). BrainVoyager provided the experimenter with visual feedback of the 3D distance between a vector through the hotspot (perpendicular to the center of the figure-of-eight coil surface) and any target point identified on the MRI images. The feedback was provided on a head-mounted display (Argo PC/3D, 640 × 480 pixels, 32° field of view), to ensure that the testing room remained dark throughout the trials.

Before testing in the behavioral sessions, we acquired individual MRI scans using a 1.5T scanner (Avanto, Siemens Medical Solutions, Erlangen, Germany; voxel size 1.5 mm) at Toronto's SickKids Hospital. In BrainVoyager, the MRI images were aligned with the axis from the *anterior* to the *posterior commissure* (AC-PC). We selected two target sites: MT+/V5 and SPOC. Both were first localized according to individually determined anatomical landmarks.

**Figure 1:**
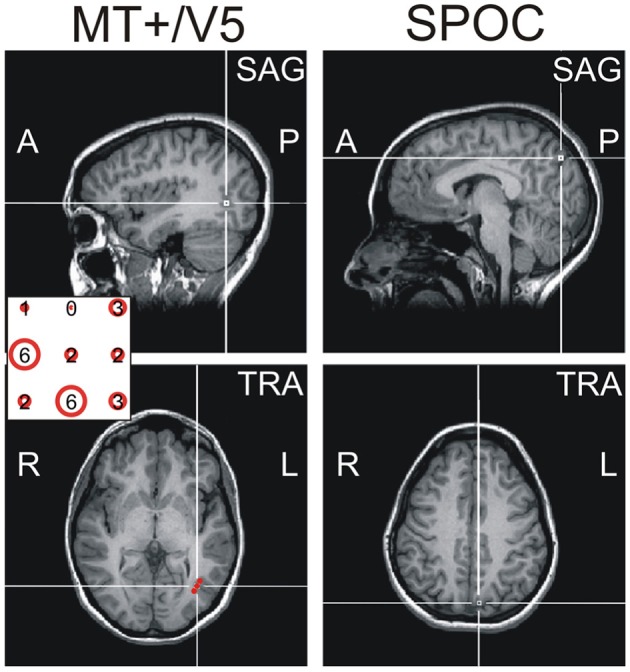
**Exemplary sagittal (SAG, top) and transversal (TRA, bottom) views of the MRI of one participant, illustrating the functional location of MT+/V5 (left) and the anatomical location of SPOC (right) using cross-hairs.** The inset for the MT+/V5 images illustrates, for all nine grid points (whose top view is illustrated in sagittal view), the difference between trials with and trials without TMS in direction judgments during the functional localizer. As can be seen, the largest effect for this participant was found for the left-center and middle-bottom points. For the left-center point, however, this effect was mainly caused by deviating performance in the trials without TMS; for this reason, we identified the middle-bottom point as the functional MT+/V5 for this participant. For most participants, a single point could be identified where the TMS effect was largest.

Specifically, we estimated MT+/V5 to be located between the intersections of the ascending limb of the inferior temporal sulcus with the lateral occipital sulcus and the inferior temporal sulcus (Dumoulin et al., 2000; Table 1). This estimate was then refined using a functional TMS-localizer involving a motion direction judgement task with random dot kinematograms (see “Procedure” details below). On the AC-PC-aligned MRI scan we created a vertical 3 × 3 grid of target points for TMS (4 voxels between grid points), which was centered on the anatomical estimate and rotated 30° around a vertical axis to align with the skull surface (Figure 1). Each participant's functional MT+/V5 was defined as the grid point that induced the largest TMS effect on motion direction judgments. Both the anatomically and functionally defined locations of MT+/V5 were consistent with previous reports (Tootell et al., 1995; Dumoulin et al., 2000; Lechak and Leber, 2012; see Table 1).

**Table 1:** **Talairach coordinates of the relevant target areas for all participants**.

	**Anatomical MT+/V5**	**Functional MT+/V5**	**Posterior MT+/V5 (Control site for MT+/V5)**	**Anatomical SPOC**
S1	(−46, −63, −4)	(−47, −59, 1)	(−41, −68, 1)	(−8, −76, 35)
S2	(−44, −70, 3)	(−43, −68, −1)	(−38, −78, −1)	(−5, −78, 24)
S3	(−44, −64, 4)	(−47, −59, 9)	(−42, −69, 9)	(−7, −76, 35)
S4	(−39, −63, 6)	(−35, −59, 0)	(−30, −67, 0)	(−5, −73, 38)
S5	(−43, −65, 9)	(−43, −65, 9)	(−38, −73, 9)	(−5, −77, 38)
S6	(−39, −71, 0)	(−39, −71, 0)	(−33, −80, 0)	(−7, −79, 23)
S7	(−39, −76, −4)	(−39, −75, −8)	(−34, −84, −8)	(−8, −80, 36)
mean(SD)	[−42(3), −67(5), 2(5)]	[−42(4), −65(6), 1(6)]	[−37(4), −74(7), 1(6)]	[−6(1), −77(2), 33(6)]

Note: coordinates provided in the format (x, y, z).

SPOC was defined as a region situated along the medial surface of the parietal lobe, medial to the intraparietal sulcus, anterior to the parieto-occipital sulcus, and posterior to the subparietal sulcus; this region includes generally the superior end of the parieto-occipital sulcus, as well as regions immediately anterior (in the precuneus) and posterior (in the cuneus) to the sulcus (Vesia et al., 2010, 2013). This corresponds to a tightly clustered reaching region described in other functional neuroimaging studies (Beurze et al., 2007; Filimon et al., 2009; Gallivan et al., 2009, 2011; Cavina-Pratesi et al., 2010; Galletti et al., 2012; see Vesia and Crawford, 2012 for review), which was most likely targeted by the rTMS currents in all participants (Wagner et al., 2009).

Similar to our previous work (Vesia et al., 2010), two additional control conditions were included to yield estimates of non-specific effects of repetitive TMS (rTMS). First, we assessed performance after stimulation of the vertex (Cz according to the 10–20 EEG coordinate system). Specifically, the vertex was defined as a point midway between the inion and nasion and equidistant from the left and right intertragal notches. Second, given MT+/V5's proximity to the ear, we conducted “sham” trials in which the coil was held close to the subject's scalp surface, but angled away so that no current was induced in the brain to control for the auditory sensations (i.e., loud clicking sounds produced by TMS).

Even though we adjusted the coil orientation for MT+/V5 to minimize stimulation of nerves and neck muscles, several of our participants reported that rTMS to MT+/V5 caused a slight discomfort and, in turn, differed from Sham and Cz stimulation. We therefore opted to use a different control site for MT+/V5, ~1.5 cm posterior to its functional location (i.e., 10 voxels posterior along the aforementioned grid; see Table 1). This location was selected because the discomfort associated with its stimulation best matched MT+/V5 in a pilot test with several participants. When asked after the actual experiment, our participants were unable to distinguish this site from MT+/V5 in terms of the discomfort experienced. Cz remained the control site for SPOC (i.e., both these sites induced negligible discomfort).

### TMS protocol

In the main experiment, we applied rTMS [10 Hz, 5 pulses (500 ms)] using a MagStim Rapid2 stimulator and a 70 mm Dual Air Film coil that was suspended above the participant's head using a nylon string connected to a counter weight. The experimenter manually held the coil at the location of interest. Stimulation parameters were well within the safety limits (Wassermann, 1998; Machii et al., 2006; Rossi et al., 2009). Duration of a trial was 3.6 s followed by a 3 s intertrial interval. Both participant and experimenters wore ear plugs to dampen the noise generated by the discharge of the TMS coil.

The simulation level used at all cortical sites was set to 110% of the participant's resting motor threshold (rMT), determined at the start of each session (110%: mean(SD) = 57.1(6.6)% maximum stimulator output). The rMT was defined as the lowest single-pulse TMS intensity over the hand area of the left primary motor cortex that resulted in a visible finger movement (i.e., muscle twitch) in the right index finger in 3 out of 6 cases (Rossini et al., 1994). To determine rMT, we first positioned the coil based on the individual's anatomical “hand knob area” (Yousry et al., 1997) and then systematically repositioned the coil or reduced stimulation intensity to find the optimal motor “hot spot.” Generally, this took less than 10 min.

### Procedure

#### Functional localizer task

Trials in the motion direction judgment task started with a fixation cross shown for 500 ms at screen center. Subsequently, a random dot kinematogram (100 dots, 343 × 343 pixels, ~12 × 12° of visual angle) was presented for five frames (59 ms) in the right visual hemifield [center of patch 343 pixels (~12°) right of the screen center]. In TMS trials only, single-pulse TMS was applied 125 ms after the random dot kinematogram appeared (Hotson and Anand, 1999). The fixation cross disappeared 500 ms later. Participants then judged the dominant motion direction of the dots by pressing the left or right button on a button box (i.e., total trial duration was 1184 ms plus the time taken to make the judgment). The next trial started 500 ms after the button press.

Participants took part in a preliminary session to determine their baseline performance without TMS with 12 repetitions of coherence levels (±99, ±80, ±58, ±42, ±32, ±23, ±15, ±8, and ±2%; negative sign denotes leftward motion). The resulting 216 trials were presented in a randomized order over four blocks. We then fit a psychometric function through the fraction of rightward judgments as a function of the level of coherence. From this function, we selected the coherence level resulting in 75% rightward judgments [mean(SD) = 0.31(0.07)]. The functional localizer task for each grid point included trials with and without TMS (10 trials each) for both leftward and rightward motion at this coherence level, as well as 40 trials without TMS with randomly selected intermediate coherence levels. All trials were presented in random order in two blocks of 40 trials. For each of the nine grid points, presented in random order, the TMS effect was quantified using the average absolute difference in rightward responses between trials with and without TMS (for the particular experimental coherence level); the point with the largest TMS effect was defined as the functional MT+/V5.

#### Interception task

**Figure 2:**
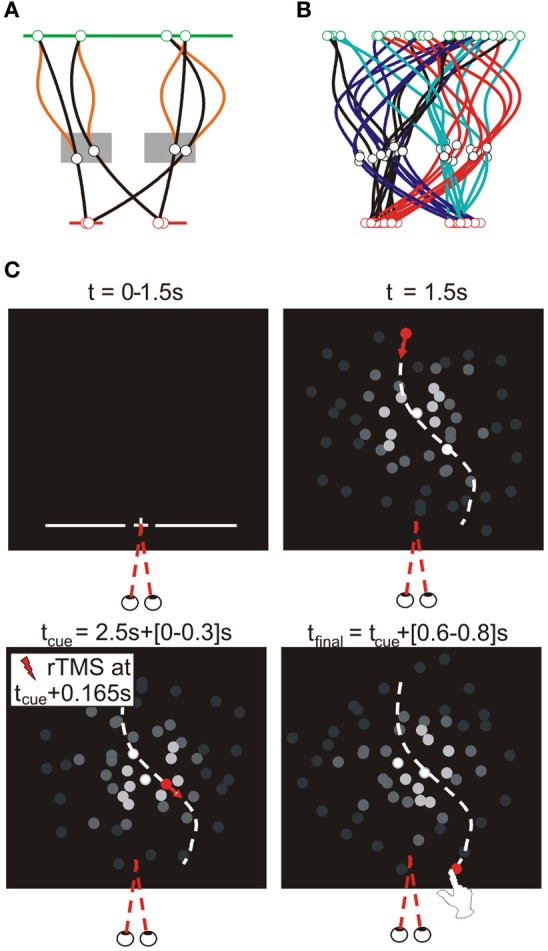
**Target trajectories.** Target trajectories were generated using the procedure illustrated in **(A)**. The target appeared 100 pixels below the upper screen edge at a random horizontal position within the central 1000 pixels of the screen (green line). A via point was selected in a zone 80–320 pixels to the left or right of screen center, 538–670 pixels below the upper screen edge (gray rectangles). The final target position was 900 pixels below the upper screen edge and randomly selected within 120–280 pixels to the left or right of screen center (red lines). Target moved downward at a constant velocity (total movement time randomly selected from 1.6 to 2.1 s), while its lateral motion was determined by fitting a polynomial through these three horizontal positions as a function of time; to increase unpredictability of the horizontal target motion an additional deviation was added between the first two points [orange lines; α(50–50*cos*(2π*t*/*T1*)), with α randomly selected between −1 and 1, *T1* being the movement time to the via-point]. Given the allowed ranges of target motion times and of the possible via-point positions, the via-point was reached after 1.0–1.3 s, after which the final position reached after another 0.6–0.8 s. **(B)** illustrates the typical variability of target trajectories in a block, showing 8 trajectories per trajectory type (as defined by the visual hemifields of the via-point and final point), in different colors. **(C)** Trial sequence for the interception experiment. As soon as participants pressed the initial position button for 250 ms, trials started with the presentation of a fixation point (central, 900 pixels below the upper edge of the screen) for 1.5 s. Simultaneously, a line of quasirandom length (600–1000 pixels) appeared centrally at the same height for 1 s, reminding participants of the required height of interception. Upon disappearance of the fixation point the moving target appeared. A beep sounded when the target reached the via-point (i.e., 1–1.3 s later), informing participants they could initiate their reach to intercept the target at the predefined height (where the target disappeared). In TMS trials, the TMS pulses started 165 ms (14 frames) after the initiation cue.

After completing the motion direction judgment task, participants took part in the main experiment. To avoid effects of fatigue on performance, we opted to conduct the interception experiment across two separate sessions (separated by a minimum of a week; inclusion of all trials in a single-session would have taken ~5 h). They were required to reach-to-intercept a target presented on a CRT screen with their right index finger. To maximize reliance on online visual control, we presented unpredictably curved trajectories that differed for each trial and were generated according to the procedure described in Figure 2A. Our procedure included trajectories within each visual hemifield after a via-point and trajectories crossing the visual midline. Timing of the initiation cue was locked to the target's arrival to this via-point and thus determined the visual hemifield of the target around movement initiation.

Moving targets were presented on a background of 72 dots (ø: 20 pixels) that were similar in shape and color to the target. Background dots moved in a random but constant direction (lifetime: four frames, duration randomly initialized). The brightness of each dots depended on its distance (D) from the brightness center, by multiplying its RGB value with $\frac{-D}{e^{60000}}$; this prevented the dots from defining the screen edges. To prevent the brightness center from providing a stable allocentric cue, it moved smoothly, but unpredictably in the central part of the screen. Specifically, a random position was determined on a circle with a random diameter (maximally 150 pixels) for all frames and this was subsequently low pass filtered (4th-order recursive Butterworth, 8 Hz).

Participants were instructed to look at the fixation point presented at trial onset throughout the trial. Figure 2C illustrates the basic sequence of events (i.e., visual stimuli presentation). We included a variable delay after the target arrived at the interception point that corrected for variations in target motion time (i.e., the delay was 1.2 s in addition to a variable interval, ranging from 0 ms for the slowest to 500 ms for the fastest target speeds). Subsequently, the room was illuminated for 1 s to prevent dark adaptation and the next trial started after an additional 0.5 s delay. If movements were not initiated between 106 and 400 ms after the cue, or if a frame refresh was missed, trials were repeated at a random position within the remainder of the block.

During a block, the coil was held at the fixed position, while trials with and without rTMS were presented. For each trajectory type we presented eight trials with rTMS and six trials without rTMS; the resulting 56 trials were randomly distributed across two consecutive blocks (for the same rTMS site). We also included two blocks without TMS (i.e., no coil touching the head or clicking sound from TMS; *not reported here*). These resulting five rTMS conditions (i.e., 10 blocks) were presented in random order. A session started with a practice block of 32 trials without TMS, with randomly selected target trajectories. During the second session (with the rTMS conditions presented in reverse order, compared to the first session) the practice block only consisted of 16 trials. Other blocks started with two practice trials without rTMS.

As mentioned before, in light of the discomfort associated with rTMS to MT+/V5, neither Cz nor Sham rTMS were deemed appropriate controls for this site. We therefore had participants perform additional recordings, involving rTMS to the new control site, posterior to MT+/V5 (described above). Because our initial analyses of gaze fixation revealed that a considerable number of trials contained too much drift, we also included trials/blocks for the other sites in these additional recordings. The number of excluded trials, and thus the amount of additional repetitions included, varied across subjects; for some, we decided to distribute the additional trials across two separate recording sessions.

### Behavioral analyses

Eye and finger movements were analyzed using Matlab (The Mathworks, Nattick, MA). The shape of the finger tip relative to the markers on the finger was calibrated by tracing the outline of the finger tip with an Optotrak pointer (~10 times). The pointer tip position data, expressed in a coordinate system attached to the rigid body, was projected onto a plane (defined by the first two principal components) and rotated within this plane by an angle that resulted in the best fit of a variable-order 2D polynomial through this projected data [the order was determined by the experimenter (4–10), based on the realism of the fitted finger shape]. This polynomial was sampled at 0.1 mm intervals and these points were added as virtual markers to the rigid body coordinates of the finger markers; thus, the finger tip shape could be reconstructed at any point in time as long as at least three markers of the rigid body on the finger were visible.

Right eye-in-head orientation was analyzed using a custom Matlab program, which performed an automatic drift correction on the mean eye orientation attained in the 800 ms before target appearance. The drift-corrected eye traces were used to exclude trials from the analysis when the horizontal eye position deviated more than 2° from the required fixation direction between target onset and 150 ms before finger-screen contact; for the vertical eye position a 4° deviation was allowed, given that it was less critical for controlling the visual hemifield in which the target was presented. We thus rejected a relatively high number of trials (419) out of 3937 trials; an additional 613 trials were rejected because the program incorrectly accepted trials where movements were initiated outside the allowed temporal window (see above).

**Figure 3:**
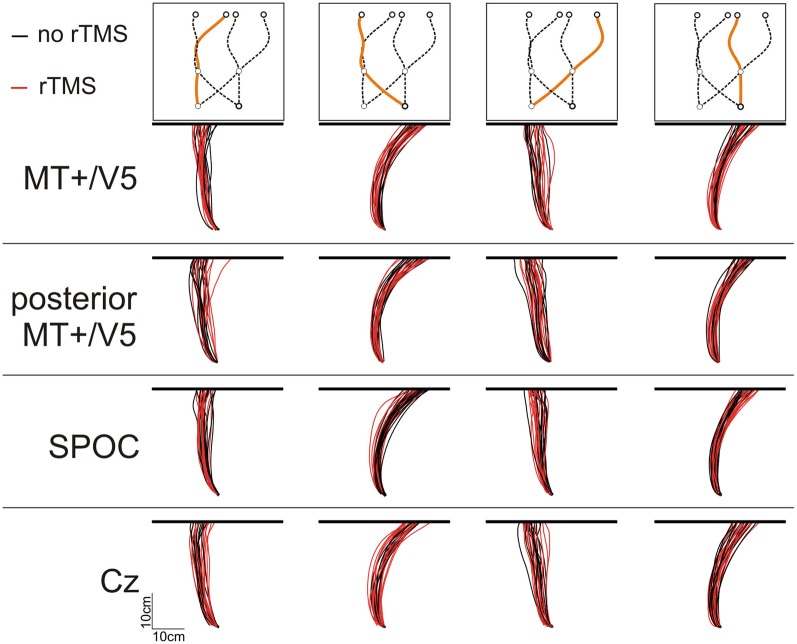
**Illustrative top views of finger tip paths of an exemplary participant for all conditions [i.e., rTMS (red lines) vs. no rTMS (black lines), four sites for rTMS (MT+/V5, posterior MT+/V5, SPOC, and Cz), and four combinations of target position at initiation and interception (illustrated above the panels)]**.

We defined finger-screen contact as the first sample at which the z-velocity of the finger tip was lower than 10 mm/s (when the finger tip was closer than 15 mm to the plastic cover). The most forward virtual point at contact (from the participant's perspective) was defined as the contact position. We calculated the trajectory of this point during the entire reach, as illustrated in Figure 3 for one participant for all conditions. We separately analyzed horizontal and vertical spatial errors; these errors were defined as the horizontal and vertical distance of finger-screen contact from the actual final target position; this definition assumes perfect timing. Temporal errors were defined as the time of finger-screen contact, relative to the time the target arrived at its final position; this definition assumes participants aimed at the actual interception height. For all three errors, we statistically analyzed the within-condition means and standard deviations (reflecting interception accuracy and precision, respectively).

### Statistical analyses

Before performing statistical analyses on the interception errors in SPSS (IBM, Armonk, NY), we averaged out the variance due to differences between sessions for each individual participant by aligning single-session data with the across-session mean. In addition, we aimed to increase the power of our final analyses by averaging out variance resulting from random variations in the target trajectory (i.e., the *x*- and *y*-position of the via-point, the horizontal final position, and the time taken for the target to move from the via-point to the final point). These factors are not independent from those in our main analyses (see below) and their associated variance thus would negatively affect the power of our main analyses. We fit a linear regression model with these four factors to the individual interception errors in each condition; hence, the data considered were this model's residuals plus its intercept.

We analyzed these interception errors in SPSS using a full factorial Site × TMS × Trajectory linear mixed model. Here, Trajectory in fact represents two factors corresponding to the visual hemifield occupied by the target at initiation and interception. In this model, each factor had a fixed (population) and random (individual) component; unique between-participant variances for all non-redundant levels of all factors were allowed (using SPSS' “Diagonal” covariance structure); degrees of freedom were obtained by SPSS using Satterthwaite's approximation. The factor Site had two levels: either MT+/V5 and posterior MT+/V5 or SPOC and Cz. We examined the effect of rTMS using linear contrasts; specifically, we tested whether the within-block effect of rTMS (difference between errors in trials with and without rTMS) was significantly different when the coil was held at the target site compared to its control site. In total, nine linear contrasts were calculated (main effect, two visual hemifields occupied at initiation, two visual hemifields at interception, and the four-associated combinations). We also assessed the significance of the 18 associated within-block rTMS effects using linear contrasts. The standard deviations were square-root transformed (Hawkins and Wixley, 1986) and analyzed using paired-samples *t*-tests (same comparisons described above). For all tests, critical *p*-values were adjusted for multiple comparisons (9 for the between-site comparisons, 18 for the within-block rTMS effects) using a step-down Holm–Sidak procedure.

## Results

In this study, we examined the role of brain areas MT+/V5 and SPOC in the spatial and temporal control of manual interception. Participants were required to reach out to intercept a target moving downwardly on a computer screen with their index finger. Target motion was unpredictable and could change at any moment during the entire trial (Figure 2); as a result, movement paths were typically curved (Figure 3), highlighting online movement updating. In each block of trials, we included trials with and without rTMS; our analyses focused on the difference between these trials, as a function of the brain site the TMS coil was aimed at. We examined the time and position of finger-screen contact, relative to when and where the target reached the pre-specified interception height. Below, we separately discuss the spatial and temporal effects of our rTMS manipulation.

### Analysis of spatial interception errors

We analyzed the interception end points, defined as the position where the finger touched the screen, relative to the final target position. Figure 3 illustrates the finger paths for an exemplary participant. Notably, we found that rTMS had a minimal effect on the horizontal spatial deviations of the interception movements, irrespective of stimulation site. Indeed, across subjects the magnitude of the horizontal errors did not differ between rTMS to MT+/V5 and its control site (all *p* > 0.075; *p*_crit_ = 0.0057) nor between stimulation to SPOC and Cz (all *p* > 0.34; *p*_crit_ = 0.0057). Further, the same pattern held for the within-block rTMS effects for MT+/V5 (all *p* > 0.05; *p*_crit_ = 0.0028) and SPOC (all *p* > 0.02; *p*_crit_ = 0.0028). Analyses of the vertical interception errors also did not reveal any significant between-site differences (MT+/V5 vs. control: all *p* > 0.16; *p*_crit_ = 0.0057; SPOC vs. Cz: all *p* > 0.085; *p*_crit_ = 0.0057). The within-block rTMS effects on the vertical errors, however, did reveal several significant differences.

**Figure 4:**
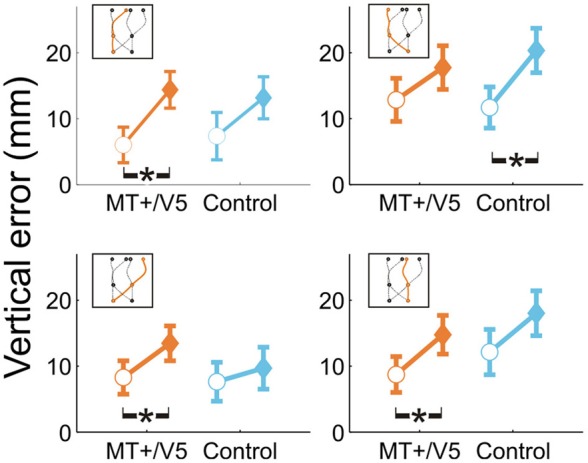
**Vertical interception errors for MT+/V5 and its posterior control site for all four trajectory types (trials with rTMS: solid diamonds; trials without rTMS: open circles).** Error bars indicate standard errors; asterisks indicate significant differences.

For all comparisons, rTMS yielded a higher interception (i.e., more positive vertical interception errors). Averaged across trajectories, the within-block rTMS effect was significant for MT+/V5 [*F*_(1, 17.8)_ = 40.1, *p* = 6 × 10^−6^; *p*_crit_ = 0.0028] and near-significant for its control site (*p* = 0.007; *p*_crit_ = 0.0057). When separated according to target position at initiation or interception, significant within-block effects of rTMS were observed for both MT+/V5 [*F*_(1, 19.4)_ = 26.5, *p* = 5.4 × 10^−4^; *p*_crit_ = 0.0032] and the control site [*F*_(1, 7.8)_ = 18.7, *p* = 0.0027; *p*_crit_ = 0.0043] for targets initially in the left visual hemifield, and for MT+/V5 for targets initially in the right visual hemifield [*F*_(1, 6.1)_ = 19.0, *p* = 0.0045; *p*_crit_ = 0.0057]. Similarly, when separated according to target position at interception, within-block effects of rTMS were only significant for MT+/V5 [*F*_(1, 19.3)_ = 29.5, *p* = 2.9 × 10^−5^; *p*_crit_ = 0.0030] for targets intercepted in the left visual hemifield, and significant for both MT+/V5 [*F*_(1, 8.0)_ = 16.4, *p* = 0.0037; *p*_crit_ = 0.0047] and the control site [*F*_(1, 10.7)_ = 20.9, *p* = 8.5 × 10^−4^; *p*_crit_ = 0.0037] for targets intercepted in the right visual hemifield. Figure 4 shows the vertical errors for all four trajectory types. For trajectories crossing from left to right the finger in fact touched the screen significantly higher only with rTMS to the control site [*F*_(1, 12.1)_ = 21.5; *p* = 5.6 × 10^−4^; *p*_crit_ = 0.0034]. However, it touched significantly higher only with rTMS to MT+/V5 if the target did not cross between the visual hemifields [left: *F*_(1, 10.6)_ = 17.7, *p* = 0.0016; *p*_crit_ = 0.0039; right: *F*_(1, 11.4)_ = 12.4, *p* = 0.0045; *p*_crit_ = 0.0051] and for targets crossing from the right to the left visual hemifield [*F*_(1, 10.5)_ = 11.8; *p* = 0.0061; *p*_crit_ = 0.0064]. Thus, overall the effect of rTMS was more consistent for MT+/V5, which is relevant for the temporal rTMS effects discussed later.

The analyses for SPOC revealed much less variations in the vertical interception errors were observed. In particular, averaged across trajectories the finger touched the screen at higher positions only with rTMS at Cz [*F*_(1, 8.9)_ = 50.12; *p* = 6.3 × 10^−5^; *p*_crit_ = 0.0037]. This held both when the target was initially located in the left [*F*_(1, 25.9)_ = 34.9; *p* = 3 × 10^−6^; *p*_crit_ = 0.0032] and right visual hemifield [*F*_(1, 6.5)_ = 23.0; *p* = 0.0024; *p*_crit_ = 0.0039], as well as for interception in the right visual hemifield [*F*_(1, 40.5)_ = 69.2; *p* < 5 × 10^−7^; *p*_crit_ = 0.0028]. Thus, rTMS to SPOC in fact did not elicit any significant variations in the height of finger-screen contact.

**Figure 5:**
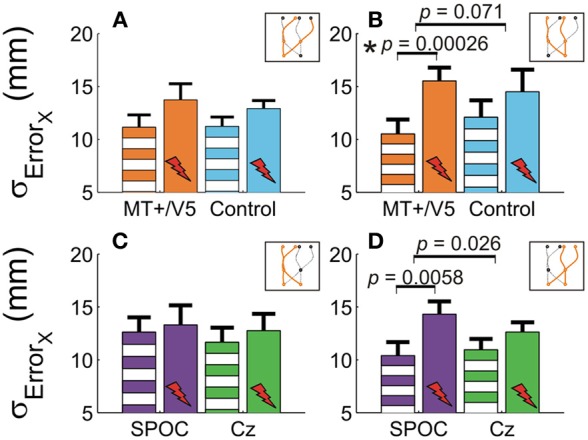
**The horizontal standard deviation of interception errors (σ_Error_*x*__) for MT+/V5 and its control site as a function of target position at interception (A and B) and for SPOC and its control site Cz as a function of target position at movement initiation (C and D).** Asterisks indicate significant differences; near-significant differences are also marked.

Our analyses of vertical spatial variability did not reveal any statistical between-site [across both sites *p* > 0.17 (*p*_crit_ = 0.0057)] and within-block differences [across all sites, *p* > 0.10 (*p*_crit_ = 0.0028)]. For the horizontal spatial variability, however, several near-significant trends are worth noting. This variability tended to increase with rTMS to MT+/V5 [*t*_(6)_ = 4.59; *p* = 0.0038; *p*_crit_ = 0.0030], but not for the control site (*p* = 0.026; *p*_crit_ = 0.0030). The magnitude of these effects, however, did not differ between the sites (*p* = 0.19). Figures 5A,B illustrate that the horizontal spatial variability only increased with rTMS to MT+/V5 for interception in the right visual hemifield [*t*_(6)_ = 7.67; *p* = 0.00026; *p*_crit_ = 0.0028], but not for its posterior control site (*p* = 0.08; *p*_crit_ = 0.0030). The comparison of the magnitude of these effects between sites, however, did not reach statistical significance (*p* = 0.071). Finally, the rTMS-induced effect at MT+/V5 for trajectories crossing from the left to the right visual hemifield tended to approach significance (*p* = 0.0178; *p*_crit_ = 0.0030), but did not for the control site (*p* = 0.69); the associated between-site difference tended toward significance (*p* = 0.0153; *p*_crit_ = 0.0028). While the effect of rTMS to MT+/V5 on horizontal spatial variability was not sufficiently pronounced to suggest a role of MT+/V5 in the spatial control of interception, a more consistent pattern emerged for SPOC.

We observed a significant within-block effect of rTMS to SPOC for the horizontal spatial variability averaged across the trajectories [*t*_(6)_ = 5.6; *p* = 0.0013; *p*_crit_ = 0.0028]. While the effect was similar in magnitude for Cz, it did not achieve statistical significance because of the larger between-participant variability (*p* = 0.13). Therefore, the difference of these effects was not significant (*p* = 0.62). Figures 5C,D depicts the effect of rTMS to SPOC and Cz for targets in the left and right visual hemifield at initiation: for targets in the right visual hemifield at initiation we found that the within-block increase horizontal spatial variability with rTMS of SPOC tended to approach significance [*t*_(6)_ = 4.18; *p* = 0.0058; *p*_crit_ = 0.0030], but not for Cz (*p* = 0.079). The difference between SPOC and Cz also tended to approach significance [*t*_(6)_ = 2.9; *p* = 0.026; *p*_crit_ = 0.0057]. The latter trend, while just failing to achieve statistical significance, is consistent with the hypothesized role of SPOC in the spatial control of interception.

In sum, only rTMS to MT+/V5 mainly induced variations in the vertical touch position, while rTMS to SPOC most consistently affected the horizontal spatial variability of the interception.

### Analysis of temporal errors

**Figure 6:**
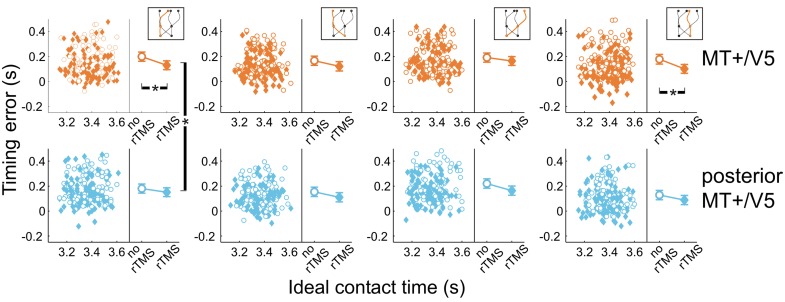
**Temporal interception errors for MT+/V5 and its posterior control site for all four trajectory types.** In the left part of each panel individual errors are shown as a function of the ideal contact time (trials with rTMS: solid diamonds; trials without rTMS: open circles); individual differences are averaged out for illustrative purposes. The right part of each panel depicts the average timing errors; error bars indicate standard errors. Asterisks indicate significant differences.

Next, we analyzed the point in time that the finger touched the screen, relative to when the target actually arrived at its final position. All temporal interception errors occurring for blocks with rTMS to MT+/V5 and its control site for the different target trajectory types are shown in Figure 6. The scatter plots show the timing errors as a function of the ideal contact time for trials with and without rTMS (which also illustrates that target movement time was randomly varied). The right side of each panel depicts the effect of TMS on the mean timing errors. This shows two general aspects of our data: (1) interception errors were generally positive, indicating that participants on average reached the screen too late; and (2) TMS resulted in an earlier interception irrespective of stimulation condition [main effect of rTMS: *F*_(1, 5.6)_ = 19.0, *p* = 0.006]. This was expected based on an earlier version of the experiment involving targets moving along a linear trajectory (Dessing et al., 2010). The critical analyses addressed whether the size of the rTMS-induced effect was larger for the target site compared to its respective control.

Figure 6 also shows that, compared to its control site, rTMS to MT+/V5 had its greatest effects on interceptive timing for the trajectories that remained in the same visual hemifield throughout movement execution. In fact, only the within-block effect of rTMS to MT+/V5 for these trajectories achieved significance [left visual hemifield: *F*_(1, 16.3)_ = 24.6; *p* = 1.4 × 10^−4^; *p*_crit_ = 0.0028; right visual hemifield: *F*_(1, 9.9)_ = 15.5; *p* = 0.0028; *p*_crit_ = 0.0034]. We also found significant differences between MT+/V5 and its control site for targets moving within the left visual hemifield [*t*_(1405.5)_ = −2.9, *p* = 0.0042; *p*_crit_ = 0.0057]. None of the other differences between MT+/V5 and its respective control site showed statistical significance.

**Figure 7:**
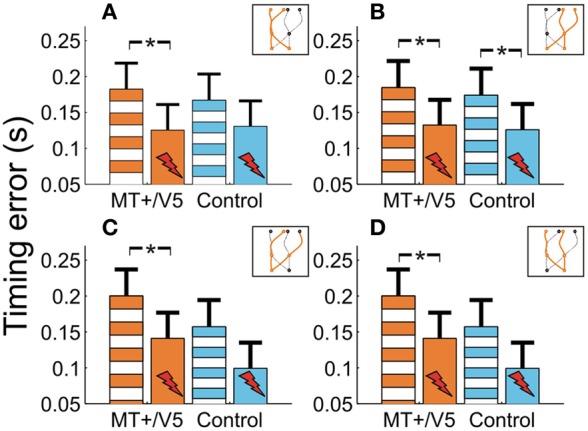
**The temporal interception errors for MT+/V5 and its control site as a function of target position at movement initiation (A and B) and as a function of target position at interception (C and D).** Asterisks indicate significant differences.

We also evaluated whether additional patterns could be discerned from the within-block rTMS effects. The within-block main effect of rTMS was only statistically significant for MT+/V5 [*F*_(1, 7.0)_ = 20.6; *p* = 0.0027; *p*_crit_ = 0.0032]. Figure 7 shows the average temporal interception errors for MT+/V5 and its control site as a function of the visual hemifield occupied by the target at movement initiation **(A,B)** or at interception **(C,D)**. We found that the within-block effect of rTMS to MT+/V5 was statistically significant for targets in the left visual hemifield at initiation only [*F*_(1, 9.6)_ = 18.0; *p* = 0.002; *p*_crit_ = 0.003], whereas for targets in the right visual hemifield at initiation the effect was significant for both sites [MT+/V5: *F*_(1, 10.5)_ = 13.7; *p* = 0.0038; *p*_crit_ = 0.0043; control site: *F*_(1, 11.4)_ = 11.4; *p* = 0.0043; *p*_crit_ = 0.0045]. When grouped according to the visual hemifield where interception occurred, only the within-block rTMS effect for MT+/V5 was significant [left: *F*_(1, 9.9)_ = 15.1; *p* = 0.0031; *p*_crit_ = 0.0039; right: *F*_(1, 9.8)_ = 15.3; *p* = 0.0030; *p*_crit_ = 0.0037]. While none of these effects yielded a significant difference between MT+/V5 and its control site, the pattern of results suggest that rTMS not only had a greater, but also a more consistent effect on interceptive timing when applied to MT+/V5.

**Figure 8:**
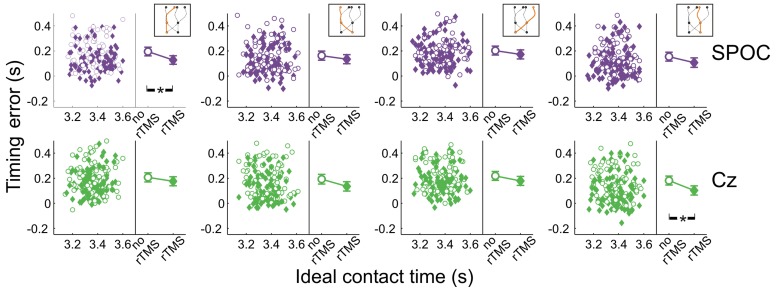
**Temporal interception errors for SPOC and its control site Cz for all four trajectory types.** In the left part of each panel individual errors are shown as a function of the ideal contact time (trials with rTMS: solid symbols; trials without rTMS: open symbols); individual differences are averaged out for illustrative purposes. The right part of each panel depicts the average timing errors; error bars indicate standard errors. Asterisks indicate significant differences.

Figure 8 shows the effects of rTMS to SPOC and Cz on interceptive timing for all target trajectory types. Again, rTMS generally resulted in earlier interception of the targets, denoted by positive values of timing errors [*F*_(1, 13.0)_ = 43.3, *p* = 0.0001]. The timing effects of rTMS to SPOC were less consistent than MT+/V5. The difference between effects for SPOC and Cz, however, did not achieve statistical significance (all *p* > 0.085).

Across trajectories, the within-block rTMS effects were significant for both SPOC [*F*_(1, 16.8)_ = 31.5; *p* = 3.3 × 10^−5^; *p*_crit_ = 0.0032] and Cz [*F*_(1, 6.9)_ = 19.2; *p* = 0.0034; *p*_crit_ = 0.0051]. We found that the within-block effect of rTMS was significant for SPOC when targets were within the right visual hemifield at initiation [*F*_(1, 8.0)_ = 29.8; *p* = 0.0006; *p*_crit_ = 0.0039; Cz: *p* = 0.021; *p*_crit_ = 0.0085] and for Cz when targets were in the left visual hemifield [*F*_(1, 7.8)_ = 24.8; *p* = 0.001; *p*_crit_ = 0.0042; SPOC: *p* = 0.014; *p*_crit_ = 0.0073]. For interception in the left visual hemifield, we found that only the within-block effect of rTMS to SPOC was statistically significant [*F*_(1, 22.0)_ = 31.1; *p* = 1.3 × 10^−5;^ *p*_crit_ = 0.0028; Cz: *p* = 0.021; *p*_crit_ = 0.010], whereas for interception in the right visual hemifield the within-block effect was significant for both sites [SPOC: *F*_(1, 17.8)_ = 11.6; *p* = 0.0032; *p*_crit_ = 0.0047; Cz: *F*_(1, 11.1)_ = 26.1; *p* = 3.3 × 10^−4^; *p*_crit_ = 0.0037]. Finally, Figure 8 shows that the within-block effect of rTMS was significant for SPOC for trajectories in the left visual hemifield [*F*_(1, 15.7)_ = 37.0; *p* = 1.7 × 10^−5^; *p*_crit_ = 0.0030] and for Cz for trajectories in the right visual hemifield [*F*_(1, 15.9)_ = 30.9; *p* = 4.5 × 10^−5^; *p*_crit_ = 0.0034]. Thus, while effects of rTMS on temporal biases were observed, these were not specifically associated with rTMS to SPOC.

Finally, our analyses of temporal precision did not reveal any differences between MT+/V5 and its control site (all *p* > 0.25); indeed, none of the within-block rTMS effects were significant (all *p* > 0.05). The same held for the differences between SPOC and Cz (all *p* > 0.092) and their within-block rTMS effects (all *p* > 0.17). Evidently, rTMS did not affect the variability of interceptive timing.

In sum, we did not observe consistent effects of rTMS to SPOC on the temporal features of interception performance, while for MT+/V5 we found specific rTMS-induced shifts in timing performance (i.e., earlier interception), particularly for targets moving in the left visual hemifield.

## Discussion

To successfully intercept external stimuli in our environment, such as catching a ball, one must move the hand accurately to the right place at the right time (Peper et al., 1994; Dessing et al., 2005, 2009). Here, we studied the neural basis of this manual interception, specifically focusing on the role of visual motion area MT+/V5 and a movement planning region in the SPOC. Using rTMS, we aimed to interfere with the underlying neural activity in these areas during the execution of a reach-to-intercept action to a target moving in a downward direction on a screen. One problem in studying interception relates to the difficulty to experimentally differentiate spatial and temporal control. We attempted to resolve this by predefining the interception location in one dimension (i.e., its height), and defining the spatial and temporal errors relative to the ideal interception location. Thus, technically our participants only needed to control the horizontal position and time at which to intercept the target.

TMS had several effects in our study—some of these were statistically significant, whereas others were only trends that approached statistical significance. Some of the rTMS-induced timing effects for MT+/V5 went hand in hand with effects on vertical interception errors (i.e., earlier = higher interception). This pattern of results, while subtle, was consistent with the notion that MT+/V5 contributes to the spatial and temporal control of interception For SPOC the effects were less robust, and only observed for the spatial dimension. We discuss these findings in more detail below.

### Temporal control of interception

In our experiment, finger-screen contact occurred about 200 ms too late on average, even without rTMS. This appears to reflect our participant's natural behavior, given our task instructions. We expected this based on a preliminary experiment (Dessing et al., 2010), but decided not to provide our participants with feedback because we did not want their performance to be diluted by learning effects. Further, rTMS consistently resulted in an even earlier finger-screen contact, irrespective of stimulation condition. This likely reflects a non-specific TMS effect, possibly via a startling effect on the movement due induced by the clicking noises associated with TMS (Tresilian and Plooy, 2006).

However, we also observed a site-specific effect, that is, a timing effect for rTMS to MT+/V5 relative to its control site for targets moving in a straight downward direction in the left visual hemifield. This appears to reflect reflects a highly site-specific functional disruption of MT+/V5, particularly because the control site was rather close to MT+/V5. The within-block effect was only significant for MT+/V5 for trajectories that not crossing between visual hemifields. However, it is possible that these effects were actually stronger and more general than what we reported, because one cannot exclude the possibility that the control rTMS may have induced small currents in MT+/V5 and/or nearby motion sensitive regions such as the lateral occipital sulcus (/kinetic occipital region; LOS/KO; Orban et al., 2003). These effects were matched by variations in the vertical interception errors, which would be expected if rTMS to MT+/V5 disrupted signals used for movement positioning and timing. Given the retinotopic organization of MT+/V5's sensitivity for visual motion (Gardner et al., 2008; Pitzalis et al., 2010)—at least for its subdivision corresponding to primate MT—the effects of rTMS to left MT+/V5 may be expected to be largely specific to the right visual hemifield. Previous studies, however, also have reported more general TMS effects for MT+/V5 on interceptive timing that were independent of the visual position of the target. For example, when participants reached and grasped receding targets that they were most likely visually pursuing (i.e., eye movements were unconstrained); rTMS to MT+/V5 decreased movement speed even though targets were predominantly foveated (Schenk et al., 2005). This appears to contradict the earlier interception we observed, but this may reflect task differences [for example, Schenk et al.'s (2005) task involved mainly target motion in depth, whereas here target motion was in the frontal plane]. Interestingly, when the fixation location was constrained, targets were judged to arrive earlier at a pre-specified position with rTMS to MT+/V5 (Bosco et al., 2008); further, this effect occurred for targets moving either vertically or horizontally (presumably within the visual hemifield encoded by the stimulated MT+/V5). Our results extend these findings and suggest that MT+/V5's involvement in interceptive timing is not organized purely retinotopically.

How does MT+/V5 contribute to interceptive timing? In humans, MT+/V5 comprises of both the middle temporal (MT) and medial superior temporal (MST) areas. MST has been associated with the computation of time-to-contact, at least for frontally approaching objects (Browning, 2012). If this extends to vertically moving targets, our findings suggest that the timing effects of stimulating MT+/V5 take place in the MST subdivision of MT+/V5. This, however, does not explain why we did not observe effects for targets crossing between visual hemifields. Moreover, the observed effects are also consistent with the possibility that target velocity signals were disrupted, and used elsewhere to control movement timing and positioning (e.g., for motion extrapolation). This underscores the need for more in depth investigations of the timing effect of rTMS to MT+/V5 during interception.

Temporal control of interception may involve a mechanism that scales movement speed prior to (Tyldesley and Whiting, 1975) or during movement (Dessing et al., 2002, 2005, 2009) to ensure that the interceptive movement is completed before or precisely when the target reaches the prospected contact point. If this mechanisms involves MT+/V5, it is unlikely to include direct influences on cortical areas associated with lateral stages of reach planning, given that MT+/V5 (i.e., neither MT nor MST, Maunsell and van Essen, 1983; Boussaoud et al., 1990) does not project directly to such areas. An indirect route via subcortical structures does exist, however. Cells encoding different aspects of expanding (i.e., frontally approaching) objects have been observed in the nucleus rotundus of pigeons (Sun and Frost, 1998; Wang and Frost, 1992)—the equivalent of aspects of the mammalian pulvinar nuclei—and the optic tectum (Wu et al., 2005)—the equivalent of the mammalian superior colliculus. These findings were recently confirmed in humans using fMRI (Billington et al., 2011). Both these areas receive signals from MT+/V5. It has been hypothesized that timing-related scaling movement speed arises through a pathway from these areas, via the basal ganglia to reach planning areas (Dessing et al., 2002, 2005).

### Spatial control of interception

While MT+/V5 is likely involved in the online visual control of reaching (Kruse et al., 2002; Oreja-Guevara et al., 2004; Dannenberg et al., 2009) and in motion extrapolation (Olson et al., 2004; Boulinguez et al., 2009; Kaas et al., 2010), its role in the spatial control of manual interception has not been examined. Our analyses of the horizontal interception errors did not strongly indicate of such a role; in fact, movement paths with and without rTMS of MT+/V5 virtually overlapped (Figure 3) and rTMS-induced effects on horizontal biases were not statistically significant. Nevertheless, rTMS significantly increased horizontal spatial variability only for MT+/V5, for targets intercepted in the right visual hemifield (Figure 7B). Moreover, as alluded to above, we observed several rTMS-induced vertical biases, which are consistent with a role of MT+/V5 in motion extrapolation. The fact that these effects only occurred in the vertical dimension may well be related to the fact that vertical target motion was more constant in our experiment. Both these effects, however, were subtle, which emphasizes that further study is needed to more precisely characterize the role of MT+/V5 in the spatial control of interception.

For SPOC none of the statistical analyses of horizontal and vertical spatial interception errors were found to be significant. This finding is in contrast with a previous study in our lab with stationary targets (Vesia et al., 2010), showing an rTMS-induced bias toward gaze for eccentric targets in the visual hemifield contralateral to the stimulated SPOC. While this may suggest that SPOC is not involved in the spatial control of interception, we believe this is at least partly due to the rather limited horizontal eccentricities of the target at initiation and interception in our experiment (i.e., ~7°); that is, for a similar range of eccentricities Vesia et al. (2010) also found a limited TMS-induced spatial bias. Consistent with Vesia et al. (2010), however, a TMS-induced increase in spatial variability was observed for SPOC if targets moved within the right visual hemifield at initiation (i.e., at the onset of rTMS).

Our previous findings indicated that SPOC encodes the reach goal position (Vesia et al., 2010), in visual space (Fernandez-Ruiz et al., 2007). Perhaps, the rTMS-induced increase in spatial variability for SPOC suggests that for manual interception SPOC relays the current, not the future gaze-centered target position. Given the subtle differences with the control site, however, further investigation is needed to support this conclusion. This most likely will require consideration of other motion-sensitive areas in the posterior parietal cortex [i.e., human homologs of the ventral intraparietal area (Colby et al., 1993), lateral intraparietal area (Fanini and Assad, 2009), V6 (Pitzalis et al., 2010), and area 7a (Merchant et al., 2004b)].

### Limitations

Several limitations should be noted when considering our interpretations. While the subtle nature of our results may be related to the somewhat small sample (*n* = 7), they were still larger than the TMS effects that we observed for MT+/V5 in a preliminary experiment (Dessing et al., 2010). While the methodology of this experiment differed slightly (i.e., linear target motion, which was invisible during the reach), the consistently modest effects suggest that TMS over MT+/V5 may have a more subtle effect for manual interception than for reaches to stationary targets (e.g., Vesia et al., 2010). Second, our results do not afford explicit comparisons between MT+/V5 and SPOC, because they had different control sites and were localized differently (anatomically vs. functionally). While such comparisons were not necessary for the questions posed in this study, the future investigations described may well require such comparisons.

Another limitation concerns the proximity of the alternative control site to MT+/V5 and other motion sensitive regions (e.g., area LOS/KO; Orban et al., 2003). Although this optimally controls for discomfort and provides a very precise estimate of site-specificity (as mentioned above), this control may have also have influenced motion processing. This may partly explain that the significant rTMS effects for the control site (Figure 4, trajectories from right to left). In spite of this, we observed timing differences between MT+/V5 and this nearby control site (see above). Several strategies may help to improve the control of discomfort in future experiments. One solution may be found in sham coils that provide electrical stimulation (i.e., discomfort) and sound comparable to that induced by TMS without the associated effects inside the skull (e.g., Rossi et al., 2007; Borckardt et al., 2008). Alternatively, functional tests [e.g., using TMS (as used here) or functional MRI] can be used to identify not only target sites but also negative effects at potential control sites.

Given that brain stimulation does not only affect the targeted local region but also activity in remote interconnected regions (possibly through induced modification of reciprocal connections within the circuit, Wagner et al., 2009), future work should consider combining brain stimulation with concurrent neuroimaging (Driver et al., 2009). Finally, caution should be exercised when drawing conclusions from negative or non-significant results in a TMS study, because it does not enable one to exclude that the region is necessary for this behavior (Chouinard and Paus, 2010); that is, certain sites and functions may not be as sensitive to TMS as others, perhaps because of redundancy in the system. Future work using neuroimaging techniques, along with TMS connectivity approaches (Rothwell, 2010; Vesia and Davare, 2011), should examine both effective and functional connectivity for these actions.

## Concluding remarks

We observed moderate effects of rTMS to the left hemisphere on both interceptive timing and positioning with the right hand. Specifically, when stimulating MT+/V5, targets were intercepted earlier and marginally higher when moving in a straight downward direction, particularly in the left visual hemifield. Stimulation to SPOC tended to increase the horizontal variability for targets positioned in the right visual hemifield at initiation. These results were not as robust as some reported in previous studies of TMS and motion processing (Hotson and Anand, 1999; Bosco et al., 2008), perhaps because of our highly conservative choice of control site, but they are among the first results pertaining to actual reach movements (see also Schenk et al., 2005). The findings tentatively point to an anatomical dissociation of spatial, temporal, and spatiotemporal control at an early stage of the visuomotor transformation for manual interception.

### Conflict of interest statement

The authors declare that the research was conducted in the absence of any commercial or financial relationships that could be construed as a potential conflict of interest.
